# Sustainable Cyclodextrin Modification and Alginate Incorporation: Viscoelastic Properties, Release Behavior, and Morphology in Bulk and Microbead Hydrogel Systems

**DOI:** 10.3390/gels11110875

**Published:** 2025-11-01

**Authors:** Maja Čič, Nejc Petek, Iztok Dogša, Andrijana Damjanović, Boštjan Genorio, Nataša Poklar Ulrih, Ilja Gasan Osojnik Črnivec

**Affiliations:** 1 National Institute of Chemistry, 1000 Ljubljana, Slovenia; maja.cic@ki.si; 2 Faculty of Chemistry and Chemical Technology, University of Ljubljana, 1000 Ljubljana, Slovenia; nejc.petek@fkkt.uni-lj.si (N.P.); bostjan.genorio@fkkt.uni-lj.si (B.G.); 3 Biotechnical Faculty, University of Ljubljana, 1000 Ljubljana, Slovenia; iztok.dogsa@bf.uni-lj.si (I.D.); natasa.poklar@bf.uni-lj.si (N.P.U.)

**Keywords:** cyclodextrin, alginate, dry modification, solvent-free, hydrogels, viscoelasticity, rheometry, microcarrier design

## Abstract

Incorporating cyclodextrins (CDs) into ionically crosslinked polysaccharide matrices offers a promising strategy for developing well-defined, safe-by-design and biocompatible carrier systems with tunable rheological properties. In this study, *β*-cyclodextrin (*β*-CD) was functionalized with citric acid (CDC) and maleic anhydride (CDM) using solvent-free synthesis to improve compatibility with alginate hydrogels. The modified CDs were characterized by FTIR, ^1^H NMR, DLS, zeta potential, and MS, confirming successful esterification (4.0 and 3.4 –OH substitution for CDC and CDM, respectively) and stable aqueous dispersion. Rheological measurements showed that native CD accelerated gelation (within approximately 30 s), while CDC and CDM delayed crosslinking (by 2 to 13 min) and reduced gel strength, narrowing the linear viscoelastic range to 0.015–0.089% strain due to competition between polycarboxylated CDs and alginate chains for Ca^2+^ ions. Vibrational prilling produced alginate microbeads with diameters of 800–1000 µm and a simultaneous increase in size and CD concentration. Hydrogels demonstrated high CD retention (>80% after 28 h) and slightly greater release of CDC and CDM than native CD. Overall, solvent-free modification of CDs with citric and maleic acids provides a sustainable approach to tailoring the gelation kinetics, viscoelasticity, and release behavior of alginate-based hydrogels, offering a versatile, food- and health-compliant platform for controlled delivery of bioactive compounds.

## 1. Introduction

In the past decade, cyclodextrins (CDs) and their derivatives have become valuable components in biomedical, pharmaceutical, environmental, and materials science applications, including controlled release, the stabilization of bioactives, and the design of sustainable soft-matter systems [1,2,3,4,5]. Cyclodextrins (CDs) have been further developed as promising candidates due to their unique molecular structure containing cyclic oligosaccharides consisting of α-(1→4)-linked glucose subunits forming a toroidal shape with a hydrophobic inner cavity and a hydrophilic outer surface. The amphiphilic architecture allows CDs to form non-covalent inclusion complexes with a variety of guest molecules, especially hydrophobic bioactive compounds [1,2,3,4,5,6,7]. These CDs complexes improve the solubility, chemical stability and bioavailability of encapsulated compounds and offer the potential for controlled release and masking of undesirable sensory properties [5,8].

**Figure 1:**
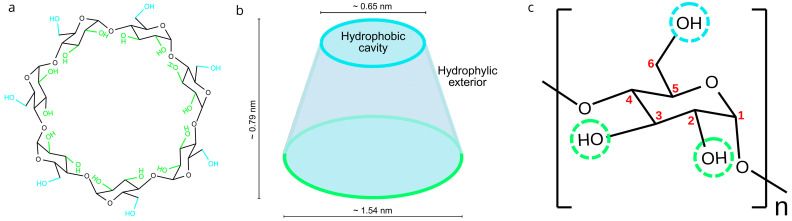
Schematic representation of (**a**) the native *β*-cyclodextrin (*β*-CD) composed of seven glucose units; (**b**) a truncated cone shape of *β*-CD; (**c**) a single glucose unit of *β*-CD (*n* = 7), with carbon atoms numbered in red and hydroxyl groups circled in green and blue dashed lines, indicating the positions available for functionalization via esterification with citric acid (CDC) or maleic acid (CDM), leading to derivatives with varying degrees of substitution (DS) (formulas prepared with ChemDraw 25).

The three naturally occurring *α*-, *β*- and *γ*-CDs are assembled with a different number of glucose units (6, 7 and 8, respectively). The number of glucose units determines the molecular and internal cavity size, the solubility and host–guest compatibility of CDs. Among these, *β*-CD is the most widely used because it balances production price, cavity size, solubility and regulatory acceptance (Figure 1).

Integrating CDs as hydrogel compounds can provide valuable functional features. Hydrogels based on natural polysaccharides such as alginate have attracted increasing attention due to their biocompatibility, mild gelation conditions, and tunable viscoelasticity [9,10,11,12]. Alginate forms hydrogels through ionic crosslinking with multivalent cations (e.g., Ca^2+^), making it suitable for biomedical, food, and environmental applications. Combining alginate with cyclodextrins (CDs) provides an opportunity to develop hybrid materials with tailored physicochemical and mechanical properties. However, the poor solubility and limited reactivity of native *β*-cyclodextrin (*β*-CD) may restrict its integration within the gel network [13].

To overcome these challenges, we propose for the CDs to be chemically modified by esterification or crosslinking with suitably safe polycarboxylic acids, such as citric acid (CA) or maleic acid (MA). Chemical modification (e.g., esterification with citric acid or maleic anhydride) increases carboxyl density, aqueous compatibility, and potential ionic or hydrogen bonding with alginate chains, thereby improving network integration beyond simple inclusion complexation [14,15]. Previous studies have shown that esterification with CA or MA enhances aqueous compatibility and introduces reactive functionalities suitable for gel network formation [16,17,18,19].

Incorporating modified CDs into alginate matrices can improve gel strength, and structural stability. Although several studies have examined CD–polymer systems, a quantitative understanding of how chemical modification of CDs affects gelation kinetics, viscoelasticity, and retention behavior remains limited [20,21,22,23].

Recent reviews have highlighted advances in cyclodextrin-based materials and sustainable CD–acid functionalization strategies [1,2,3,6,7,14,15]; however, systematic correlations between the degree of substitution (DS), alginate composition (M/G ratio as a determinant of gel rigidity), viscoelastic properties (G′), and release characteristics are still lacking.

Recent reviews highlight advances in cyclodextrin inclusion technologies and CD–citric acid–based materials [1,2,3,6,7,14,15]; however, a consolidated and quantitative understanding linking the degree of substitution (DS) of modified CDs, alginate composition (M/G ratio as a determinant of gel rigidity), viscoelastic parameters (G′), and release behavior is still lacking. Furthermore, due to existing complex protocols for the modification of CDs and/or for CDs gelation, development of simpler, safer and cost-effective procedures is required. The present study addresses these gaps.

Solvent-free modification of *β*-CD with CA and MA was investigated to enable efficient incorporation into alginate-based hydrogels. Two derivatives, citric-acid-modified *β*-cyclodextrin (CDC) and maleic-acid-modified *β*-cyclodextrin (CDM), were synthesized to introduce new functional groups that modify their interaction with alginate. The effects of these derivatives, applied at various concentrations and in combination with alginate, were evaluated to determine how chemical modification influences gelling dynamics and structural properties. Rheological techniques, optical microscopy, and spectroscopic methods (UV–VIS, FTIR, NMR, and MS) were used to characterize the systems.

The overall aim is to explore how these green, simple modification routes affect gelation dynamics, network mechanics, and CD retention, thereby establishing a structure–function framework for the rational design of tunable, sustainable biohydrogels for use in biocompatible materials, controlled release systems, and soft-matter engineering.

## 2. Results and Discussion

### 2.1. Functionalization of β-CDs with Maleic Acid

To establish a benchmark for cyclodextrin functionalization, *β*-CD was first modified with maleic anhydride using a conventional solvent-based method. This reference approach enabled comparison with the newly developed solvent-free synthesis in terms of substitution efficiency, purity, and downstream applicability in hydrogels.

The modification procedure involved the functionalization of *β*-Cyclodextrin (*β*-CD) with maleic anhydride (1:12 molar ratio CD:MA and 6 h reaction time), which was initially conducted at 70 °C using anhydrous dimethylformamide (DMF) solvent and considered as reference. A good degree of substitution (DS) of 3.2 was achieved and approximated by ^1^H NMR. It is noteworthy that the isolated product still contains significant amounts of impurities such as DMF.

The presence of residual DMF and other impurities may also alter the surface chemistry and solubility of the modified CD, potentially interfering with ionic crosslinking and network formation in alginate hydrogels. Moreover, because DMF is a toxic solvent, its incomplete removal raises biocompatibility concerns [24,25,26], providing an additional rationale for developing solvent-free mechanochemical modification routes.

To prevent the contamination of the final product with the toxic anhydrous solvent and to avoid the use of heavily regulated DMF, a dry method for the functionalization of *β*-CD with maleic anhydride was developed through mechanochemical activation by employing ball milling.

The resulting product had significantly improved purity and a comparable substitution efficiency with a DS of 3.4 determined by ^1^H NMR [27,28], proving that this method is more efficient and favorable for further use.

Although the difference in DS values between the dry and solvent-based methods was modest (3.4 vs. 3.2), it indicates a more complete substitution of hydroxyl groups, providing additional carboxyl functionalities that may enhance subsequent ionic interactions in the hydrogel system.

Overall, solvent-free mechanochemical modification produced a purer product with comparable or slightly higher substitution, demonstrating an efficient and sustainable route suitable for incorporation into biocompatible gels.

### 2.2. Functionalization of β-CDs with Citric Acid

Following the maleate model, *β*-CD was further modified with citric acid to introduce multi-carboxyl functionality under solvent-free conditions, aiming to enhance aqueous compatibility and ionic interactions with alginate chains.

During the dry modification process, CD was functionalized with CA in different molar ratios (CA:CD = 2:1, 1:1 and 0.5:1) and reaction time (10 min and 30 min). Functionalization was performed in the solid state by heating the mixture in an oven at various temperatures (100–140 °C). Mass balances indicated material losses ranging from 10% to 40%, primarily attributed to losses during transfer steps and side reactions, including water formation and the generation of non-solid or low molecular weight by-products. Dialysis of the reaction mixture showed that about 30% of the initial mass was recovered in the first filtrate, which is consistent with values reported in the literature [17], while subsequent filtration steps made only a minor contribution and were thus omitted in further experiments.

The majority (98%) of the reaction products remained water-soluble. The dialyzed retentates contained higher molecular weight species (>3000 Da) corresponding to unmodified CDs, modified CDs and potential CD dimers.

Varying the reaction temperature (100–140 °C) had only a limited effect on the mass balance above 120 °C, although higher temperatures and simplified processing (fewer purification steps) resulted in more consistent yields. Based on these results, the following optimal conditions were selected for further work: 1:2 molar ratio of CD:CA, 140 °C reaction temperature and 30 min duration, with a single centrifugation and dialysis step to minimize complexity and material loss.

In all cases, the degree of substitution and extent of functionalization could not have been deduced from the yield alone and was further investigated using spectroscopic techniques. Results indicate an expected trade-off between reaction severity and product yield, suggesting that optimal conditions prioritize achieving the desired degree of substitution over maximizing yield. Namely, a higher degree of substitution can potentially improve functional properties, and an excessive modification could compromise solubility and material performance [29].

Optimization confirmed that a 2:1 CA:CD molar ratio, 140 °C, and 30 min provide efficient esterification with minimal material loss, yielding highly soluble products suitable for further physicochemical and structural characterization.

#### 2.2.1. Particle Size and Zeta Potential

To assess whether the dry modification affects the supramolecular integrity and colloidal stability of *β*-CD, we performed particle size and zeta potential analyses.

Dynamic light scattering (DLS) was used to determine the hydrodynamic diameter (Figure S1) and zeta potential of the dialyzed filtrates (Table S1).

For the sample with molar ratio 1:2 molar ratio of CD:CA and 30 min reaction time, the particle size distribution showed an average hydrodynamic diameter of about 1.38 nm, which corresponds to the expected size of a single CD molecule [30]. A high polydispersity index (PDI > 0.4) was observed, indicating good size heterogeneity, within the monomeric range. Although a PDI value above 0.4 typically indicates a moderate size distribution in colloidal systems, such values are acceptable for small molecules and supramolecular assemblies like cyclodextrins, where the measured signal reflects the Brownian motion of individual monomeric species rather than aggregated nanoparticles [6,7]. This is also reflected in a well-defined single peak in the size distribution (Figure S1), indicating uniform particle dimensions and the absence of additional individual peaks at larger hydrodynamic diameters, which would otherwise suggest aggregation. The higher PDI likely results from a combination of (a) low-intensity scattering near the DLS resolution limit for approximately 1–2 nm species, which inflates cumulant-based PdI values; (b) charge heterogeneity introduced by higher carboxyl substitution (broader mobility distribution); and (c) minor low-Mw by-products detectable in filtrate fractions. Together, these factors increase apparent heterogeneity without shifting the mean hydrodynamic diameter beyond that of monomeric *β*-CD, consistent with the unmodified CD showing no ester band and successful ester formation in modified samples [31].

Since conjugation of CA to *β*-CD is unlikely to significantly change the molecular size, DLS analysis cannot directly confirm the functionalization. However, the results indicate that there was no CD-carboxylic acid polymerization or CA-CA dimer formation, indicating the presence of predominantly monomeric species in the analyzed fractions.

Zeta potential values (summarized in Supplementary Table S1) further revealed a consistently negative surface charge in all filtrate and retentate fractions, with values ranging between −5.1 mV and −28.9 mV. These results are consistent with literature values for CD; for example, −26.3 mV [32] reflects the orientation of the hydrophilic hydroxyl (OH) groups towards the aqueous environment, which explains the surface hydrophilicity of the molecule [21]. Although most of the samples exhibited moderate colloidal stability, only the samples with higher CA content approached the threshold value of −30 mV associated with increased dispersion stability [33]. The samples from the second reaction batch and those with a lower CA:CD ratio exhibited reduced zeta potential, indicating reduced colloidal stability and possible aggregation.

TheDLS analysis confirmed the predominantly monomeric nature of the modified *β*-CD products and provided a valuable insight into their colloidal behavior. Although the product exhibited hydrodynamic dimensions consistent with successful solubilization [30], the zeta potential values indicate that the surface charge, consequently stability was significantly affected by the amount of CA used in the reaction, as anticipated from attaching negatively charged species to CD.

DLS confirmed the presence of monomeric species and the absence of polymerization, while increasingly negative zeta potentials indicated enhanced surface carboxylation and charge stabilization at higher CA content.

#### 2.2.2. FTIR Analysis

FTIR spectroscopy was used to verify ester bond formation between citric acid and *β*-CD and to evaluate the effects of temperature and time on the extent of functionalization.

**Figure 2:**
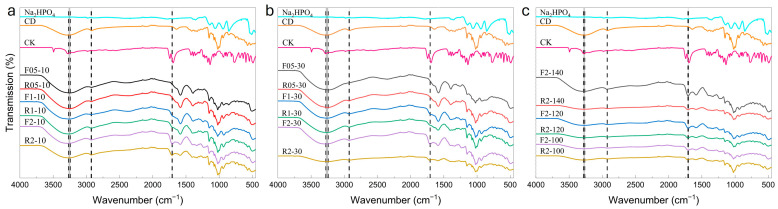
FTIR spectra of reactants and products from the dry modification of *β*-CD with CA at (**a**) 10 min reaction time. (**b**) 30 min reaction time. (**c**) varying temperatures. Product labels “F” and “R” refer to filtrate and retentate fractions, respectively. The first number in the sample code indicates the CA:CD molar ratio (X:1), and the second number corresponds to the reaction time at (**a**,**b**) 140 °C in min or (**c**) after 30 min at temperature in °C. The spectra from top to bottom: first for the pure reagents, followed by the modified samples. Characteristic peaks are highlighted with dashed lines.

FTIR spectroscopy was used to analyze the raw reagents and the synthesized products in both filtrates and retentates, as shown in Figure 2.

Key absorption bands were observed at ~3242–3284 cm^−1^ corresponding to hydroxyl (O–H) stretching vibrations. The IR bands at ~2924–2928 cm^−1^ are assigned to aliphatic C–H bonds, and those at~1704–1710 cm^−1^ are indicative of ester-carbonyl (C=O) stretching [29]. The IR band for C=O is of particular importance as it indicates the successful esterification reaction between the carboxyl groups of CA and the hydroxyl groups of the CD.

The spectra of the starting materials (CA, CD and catalyst) showed no ester band in the unreacted CD, while characteristic hydroxyl and C–H bands were present. For CA, additional peaks were observed near 1694 and 1743 cm^−1^, representing the asymmetric and symmetric C=O stretching, respectively, of the free carboxylic acids. In contrast, the esterified CDC samples exhibited an additional absorption band in the expected range (~1704–1710 cm^−1^), confirming the formation of covalent bonds, similarly reported previously for CD-CA polymerization [16,34].

Initially, as shown in Figure 2a, only two samples (F2-10 and R2-10) showed notable ester peaks, with a more pronounced band observed in the filtrate’s spectrum, indicating a partial reaction. Figure 2b presents that at higher CD concentrations, two samples (F2-30 and R2-30) showed similar trends, with the ester peak in F2-30 being more pronounced. At higher temperatures series (Figure 2c), i.e., 140 °C, all samples showed distinct ester bands, with the most intense absorbance observed for F2-140, indicating the highest degree of esterification under current conditions.

Results indicate that increasing the reaction temperature and time promotes the formation of ester bonds, suggesting improved efficiency of CD functionalization, whilst preventing dimerization or polymerization (absence of characteristic peaks). Under these conditions, the FTIR spectra showed the most intense ester C=O band, confirming the optimal degree of substitution and yield. Therefore, the FTIR results confirm that a molar ratio of 2:1 for CA:CD and a reaction temperature of 140 °C are optimal to achieve effective CD modification. Considering these factors, the F2-30 sample prepared under these conditions was selected for further use in subsequent hydrogel formulations.

#### 2.2.3. ^1^H NMR Analysis

^1^H NMR spectroscopy was used to further confirm the formation of covalent bonds between *β*-CD and CA or MA and to determine the substitution ratio. The NMR spectra were recorded in deuterium oxide on a Bruker Ascend neo NMR 600 instrument (Bruker, Billerica, MA, USA) at 600 MHz for ^1^H and processed using MestReNova software (version 14.3.1, Mestrelab Research, Santiago de Compostela, Spain).

Figure S2a displays the ^1^H spectrum of CA-modified *β*-CD. Analysis revealed that, in addition to the protons of the *β*-CD ring (3.53–4.01 ppm), additional doublet signals at 2.86 and 2.76 ppm were assigned to methylene protons of the CA moiety, respectively, which confirms the formation of the *β*-CD–CA conjugate (CDC) and is consistent with literature reports [35]. Quantitative NMR analysis was obtained from the ratio of methylene protons to the seven H1 protons of the *β*-CD ring (5.06 ppm) and revealed a substitution ratio of approximately 4.0 CA moieties per *β*-CD ring on average.

The ^1^H spectra of MA-modified *β*-CD are shown in Figure S2b,c. Figure S2b shows the ^1^H NMR spectrum of the product obtained via solvent-based method. In addition to the protons of the *β*-CD ring (3.43–4.50 ppm), clear signals at 6.48 and 6.25 ppm were assigned to the vinyl protons of the MA moiety, which confirms the formation of the *β*-CD–MA conjugate (CDM) and is consistent with literature reports [18,36]. Quantitative NMR analysis was obtained from the ratio of vinyl protons to the seven H1 protons of the *β*-CD ring (5.09 ppm) and revealed a substitution ratio of approximately 3.2 MA moieties per *β*-CD ring on average. Figure S2c shows the ^1^H NMR spectrum of the product obtained via solvent-free method. In addition to protons of the *β*-CD ring (3.43–4.68 ppm), clear signals at 6.51 and 6.31 ppm were assigned to the vinyl protons of the MA moiety. Quantitative NMR analysis was obtained from the ratio of vinyl protons to the seven H1 protons (5.03 ppm) and revealed a substitution ratio of approximately 3.4 MA moieties per *β*-CD ring on average.

Distinct methylene and vinyl proton signals verified successful esterification, with DS values of approximately 4.0 for CDC and approximately 3.4 for CDM, demonstrating comparable substitution efficiency for both modification strategies.

#### 2.2.4. Selection of Modified CDs for Gel Incorporation

Based on combined spectroscopic and physicochemical data, the most promising CD derivatives were identified for incorporation into alginate hydrogels.

The *β*-CD samples, developed with a molar ratio of 2:1 for CA:CD at 140 °C for a 30 min reaction, were chosen for further incorporation into the hydrogel based on FTIR, ^1^H-NMR, MS (refer to Supplementary Materials), and physicochemical characterization, which includes mass balance, DLS, and zeta potential analysis. These samples, especially F2-30 exhibited a favorable combination of efficient functionalization. FTIR spectra showed the most intense ester bond under these conditions, confirming the optimal degree of substitution and yield. The overall isolated yield was 66% for CDM (dry method), and the water-soluble fraction for CDC was 98%. The products also exhibited good colloidal stability in aqueous media, making them suitable for further gel formulations.

CDM was used as a reference sample to evaluate the influence of another dicarboxylic acid on the hydrogel properties. ^1^H-NMR confirmed the successful conjugation of MA to CD (three substitutions per CD ring), and the presence of vinyl groups suggests a potential for additional crosslinking or functional reactivity within the hydrogel matrices. Although MA was not the primary modifier, CDM provides additional information about structure–function relationships in differently substituted CD systems.

Samples with high purity, good colloidal stability, and controlled DS (notably F2-30) were selected to ensure a representative comparison between CDC, CDM, and native CD in subsequent gelation studies.

### 2.3. Characterization of Bulk Gels

The integration of modified CDs into alginate matrices was investigated to determine how molecular functionalization affects gelation kinetics, viscoelasticity, and structural integrity.

Hydrogels were prepared by incorporating CA-modified and MA-modified *β*-CD into aqueous alginate solutions. Preliminary experiments focused on testing different types of alginates (low and medium viscosity sodium alginate) and optimizing the gelation conditions. The hydrogels were formed by ionic crosslinking with calcium chloride (CaCl_2_) as a gelling agent.

A standardized protocol was developed in which modified cyclodextrins were incorporated into 2% (*w*/*v*) sodium alginate solutions at concentrations of 0.1%, 0.2%, and 0.5% (*w*/*v*) prior to gelation. These concentrations were chosen to evaluate the influence of CD content on viscosity and gelling behavior. For hydrogel formation, an optimized formulation containing 2% alginate and 5% (*w*/*v*) modified CD was used, with gelation induced by immersing in 0.1 M CaCl_2_ for 30 min. These concentrations ensured structural integrity and homogeneous crosslinking without syneresis or phase separation. These conditions provided reproducible gel formation and sufficient structural integrity for subsequent physicochemical evaluation.

Modified CDs changed gelling dynamics and rigidity in a concentration-dependent manner, confirming that chemical substitution directly affects macroscopic network properties.

#### 2.3.1. Viscoelastic Gelation Studies

Rheological tests were conducted to monitor time-dependent gel formation and mechanical behavior, linking CD structure to network strength.

**Figure 3:**
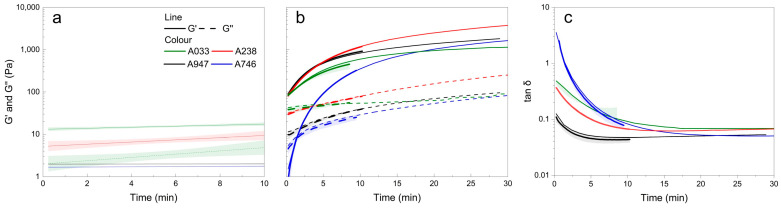
Rheological properties of four different sodium alginate solutions. (**a**) Storage modulus (G′) and loss modulus (G″) of 2% alginate solutions without the addition of crosslinker (GDL) are plotted on a logarithmic scale. Linear regression curves with 95% confidence intervals are shown to illustrate the viscoelastic development over time. (**b**) Time-dependent evolution of G′ and G″ during internal gelation of alginate hydrogels. (**c**) Time-dependent evolution of loss factor (tan δ) during internal gelation of alginate hydrogels. Thicker lines represent average values with shadowed area for standard deviations; thinner lines represent individual replicate measurements. Values below 1 Pa are not shown on the *y*-axis for G′ and G″, as they fall below the reliable detection limit.

**Figure 4:**
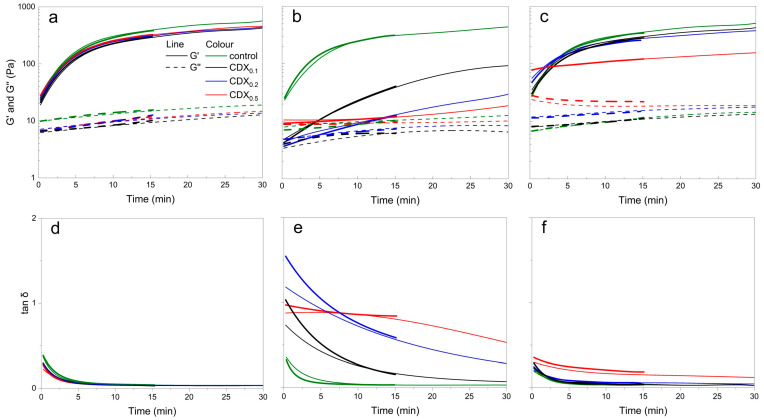
Time-dependent evolution of storage modulus (G′), loss modulus (G″), and loss factor (tan δ), plotted on a logarithmic scale, for 1.5% alginate hydrogels containing different concentrations of differently modified CDs (CDX), where X is of (**a**,**d**) native CD, (**b**,**e**) CDC, and (**c**,**f**) CDM, crosslinked with GDL and CaCO_3_. Thinner lines represent individual replicates, while thicker lines correspond to averaged data. Curves were smoothed with adjacent averaging and fitted with 2nd–5th order polynomials to visualize overall trends.

**Figure 5:**
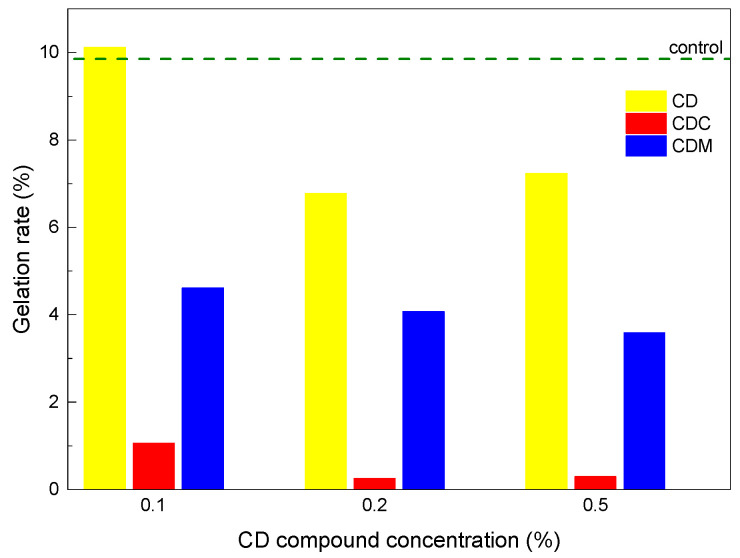
Rate of gelation after 15 min as function of CD derivative concentration.

To evaluate time-dependent viscoelastic behavior, oscillatory measurements with constant amplitude and oscillation frequency were performed (Figure 3, Figure 4 and Figure 5). Firstly, hydrogels were prepared from 2% solutions with different alginate types without incorporating CDs, and rheological properties were characterized beforehand (Figure 3a) and during gelation (Figure 3b,c).

Four different types of alginates from Sigma Aldrich (Waltham, MA, USA) were tested: A238 (71238, 1% 100–200 cP), A947 (180947, 1% 15–25 cP), A033 (A2033, 2% >2000 cP) and A158 (A2158, 2% 100–300 cP). A238 and A033 are classified as medium viscosity alginates, while A947 and A158 have a lower viscosity, and this differentiation was also clearly confirmed by our rheological measurements [37].

Figure 3a shows the viscoelastic properties of alginate solutions in the absence of the crosslinking agent glucono-*δ*-lactone (GDL). The highest storage modulus (G′), indicating the strongest elastic behavior, was observed for A033, followed by A238, while A947 and A158 showed similarly low G′ values. A similar trend was observed for the loss modulus (G″), with A033 showing pronounced viscous contributions, and negligible G″ values for A947, A158 and A238.

Gelation was triggered by the addition of calcium carbonate (CaCO_3_) particles to the alginate solution, followed by the addition of GDL (Figure 3b). For slow gelling sample A158, the gel point (G′ = G″) could still be observed, whereas, for the other three samples, due to rapid gelling occurring before the start of the measurement, no crossover point could be detected. After 10 min of gelation, A238 developed the highest G′, indicating the most rigid network, followed by A947, A033 and A158. The observed order of rigidity correlates with solution viscosity, as higher molecular weight and an optimal M/G ratio increase the elastic response [38].

The loss factor (tan δ) represents the quotient between G″ and G′, or the ratio between the viscous and elastic parts of the sample. High values indicate that the material is viscous, while low values indicate that the material is elastic [39]. For all samples, tan δ decreased the most during the first 5 min of gelation (Figure 3c).

The observed differences in gelling behavior and final network rigidity are consistent with the influence of guluronic acid content, which contributes to stronger gel networks. In addition, alginate chains with higher molecular weight tend to form more entangled networks and require more time to fully crosslink, resulting in higher gel strength [40]. Polymer chains with a higher number of interactions (e.g., hydrogen bonds, chain entanglement) and more robust network formation generally have a higher G’ after 10 min, however this can be affected by the rate of GDL hydrolysis, which is dependent on its initial concentration [41].

According to [42], among the alginates used in this study, A033 has the highest molecular weight (495 kDa, M/G ratio 1.9:1), followed by A238 (427 kDa, M/G ratio 0.7:1) and A947 (253 kDa, M/G ratio 1:1). These values agree with our observations, particularly regarding the guluronic acid content, with A238 containing the highest amount (60%), followed by A947 (51%) and A033 (34%), which correlates well with the gel strength observed after 10 min.

Further experiments were performed with alginate A033 (A2003, Sigma-Aldrich, St. Louis, CA, USA), which has the highest viscosity and molecular weight among the tested alginates, exhibiting suitable crosslinking dynamics.

##### Rheological Analysis for Alginate and CD Solutions

A rheological analysis was conducted on sodium alginate A033 solutions containing various CD compounds to assess the impact of the type and concentration of CD derivatives on the rheological properties of the base prior to gelation. Frequency sweep tests confirmed that sodium alginate solutions exhibit predominantly viscous behavior (G″ > G′) over the entire frequency range, which is consistent with polymer solutions without a crosslinked network.

The results (Figure S4) show that the addition of native CD did not significantly affect viscoelastic profiles, and all solutions remained primarily viscous with negligible G′ values. In contrast, CDC induced a concentration-dependent increase in G′ and G″. While CDM at higher concentrations (0.5%) occasionally led to a G′ that exceeded G″, indicating weak but different structuring effects even prior to crosslinking.

Overall, the measured values were very low and subject to large variability, and within the experimental deviation, the samples behaved similarly. Nevertheless, the trends suggest that chemical modification of CDs, particularly with MA, can slightly influence the rheological properties of alginate solutions prior to gelation which is expected to be more noticeable for higher CDM content in the feed.

##### Hydrogel Gelation Dynamics

To gain a better understanding of the gelling mechanism and the influence of different gel components on the formation of alginate-based hydrogels, rheological measurements were performed with respect to time and frequency as well. These techniques made it possible to evaluate: (i) viscoelastic properties during gel formation, (ii) kinetics of network development, and (iii) final strength of the gel.

Figure 4 shows the evolution of storage modulus (G′), loss modulus (G″), and loss factor (tan *δ*) over time for 1.5% alginate hydrogels containing different CD derivatives. Gelation was induced with CaCO_3_ and GDL.

Rapid gel formation was observed in all samples containing native CD. The crossover point (G′ = G″) occurred before the start of measurement, indicating that network formation had already started during sample preparation. Throughout the test, the materials exhibited gel formation behavior (G′ > G″), with values for G′ and G″ continuing to increase over time, indicating progressive crosslinking and consolidation of the polymer network. The steep initial decrease in tan *δ* values within the first 5 min confirms rapid gelation, followed by a gradual stabilization of mechanical properties over 10–15 min.

After 15 min, the final G′ values of the samples containing CD_0.1_ were comparable to the control, while the samples with CD_0.2_ and CD_0.5_ showed a slightly lower gel strength (about 3% less), indicating a minor influence of higher CD concentrations on the crosslinking efficiency (Figure 4).

In contrast, the samples containing CDC gelled more slowly. The CDC0.1 sample reached the gel point after about 2 min, while CDC0.2 and CDC0.5 gelled much later—after 11.1 and 13 min, respectively. Higher CDC concentrations appeared to delay gelation, probably due to impairment of calcium ion binding and ionic crosslink formation within the alginate matrix [43]. After 15 min, the G′ values in the CDC-containing samples reached values inherently close to their final steady-state (CDC_0.1_ ≈ 3800 Pa, CDC_0.2_ ≈ 5200 Pa, CDC_0.5_ ≈ 4300 Pa) and changed only minimally thereafter.

Samples containing CDM also showed pre-gelling behavior before measurement, with G′ > G″ throughout the test. Samples CDM_0.1_ and CDM_0.2_ gelled faster than the control samples, while CDM_0.5_ gelled the slowest. This change was probably due to higher initial viscosity and elastic resistance of CDM_0.5_. Nevertheless, all samples showed a characteristic decrease in tan δ during the first 5 min, indicating network formation.

Time course measurements further confirmed these results. After the release of calcium ions through GDL hydrolysis, mixtures with alginate and CD, transitioned from viscous to elastic, as evidenced by a sharp increase in G′. Native CD samples exhibited fastest gelation and highest final G′ values, while modified CDs (CDC and CDM) reduced both gelation speed and final gel strength, indicating incorporation into the gel structure. At higher concentrations, CDC and CDM likely competed with alginate for Ca^2+^ ions through ion crosslinking, chelation or steric hindrance, resulting in delayed gelation and lower alginate crosslinking density.

These effects indicate that modified CDs carrying carboxyl residues interact with the G-blocks of the alginate chain via weak forces (hydrogen bonds, van der Waals, hydrophobic interactions) and disrupt the formation of ionic crosslinks during early gelation. In contrast, native CD without acid species did not significantly affect network formation.

In summary, the presence of CD derivatives influences both the gelation kinetics and the final hydrogel structure. Figure 5 shows the gelation rate of each gel by comparing the G′ modulus data at 15 min with the final G′ modulus values of the gels. Compared to the control without CDs, the samples gelled the fastest in the order of CD > CDM > CDC.

These results demonstrate opportunities for fine-tuning the properties of hydrogels by choosing the appropriate type and amount of CD derivative, allowing for the development of customized systems with different viscoelastic and chemical profiles. Furthermore, it is noteworthy that relatively low concentrations of CD with respect to alginate show clear effects on hydrogel properties.

##### Hydrogel Deformation

Amplitude sweep tests were performed to evaluate the deformation behavior of hydrogels. These tests evaluate viscoelastic properties under increasing shear strain, from the linear viscoelastic (LVE) range, where the gel structure remains reversibly intact, to structural collapse and yielding [39].

**Figure 6:**
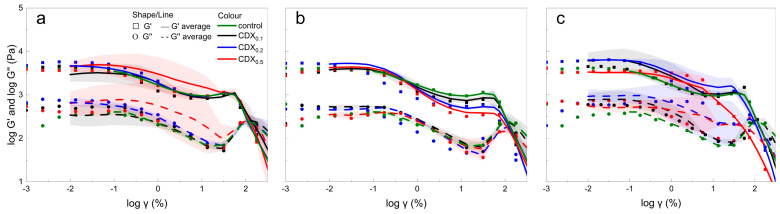
Storage modulus (G′) and loss modulus (G″), plotted on a logarithmic scale, as a function of shear strain amplitude (*γ*), presented on a logarithmic scale, for alginate hydrogels containing different concentrations of differently modified CDs (CDX), where X is: (**a**) native CD, (**b**) CDC, and (**c**) CDM. The lines represent average values with shadowed area for standard deviations and scattered points represent individual replicate measurements.

All tested samples (Figure 6) showed typical gel-like behavior within the LVE range, with G′ > G″ and both moduli remaining constant. The gel strength (G′) of all CD-containing samples with an LVE up to *γ*_c_ ≈ 0.11%, was similar to that of the control, as shown in Figure 6a. With increasing loading beyond LVE, a decrease in G′ and G″ was observed in all samples, reflecting the progressive decay of the gel network. At the crossover point (G′ = G″), G″ starts to increase due to energy dissipation as the network collapses. The height of the G″ peak was comparable for all samples, suggesting a similar level of crosslink density. The yield transition indicating the dominance of viscous behavior occurred at CD_0.1_, CD_0.2_ and CD_0.5_ between 111% and 128%, suggesting comparable structural integrity.

In Figure 6b, a similar LVE behavior was observed for CDC-containing gels, although increasing the CDC concentration slightly reduced the G″ peak heights, indicating a lower energy input for network failure. The crossover point shifted to higher strains (up to 128% for CDC_0.5_), while G′ values decreased more rapidly with increasing CDC content.

Among the CDM-containing hydrogels (Figure 6c), CDM_0.5_ exhibited the weakest structure. There was no pronounced G″ peak, and the structural collapse already occurred at *γ* = 73.8%. This state can be attributed to the effect of the initial solution’s increased viscosity on network formation. CDM_0.1_ and CDM_0.2_ showed slightly higher initial G′ values compared to the control but softened faster beyond *γ*_c_. For CDM_0.2_, a slow drop in G′ was followed by a steeper drop after 28% elongation, indicating brittle failure. The transition occurred at 111% and 97% strain for CDM_0.1_ and CDM_0.2_, respectively.

The measured critical strain values for each formulation are summarized in Table 1. All CD-containing hydrogels had relatively narrow LVE ranges. CDC-based gels had the narrowest linear range, followed by those with native CD and CDM. As the CDM concentration increased, both the LVE range and the strain range before the modulus transition shortened. A higher CD or CDC content shifted the crossover point only slightly compared to the control.

**Figure 7:**
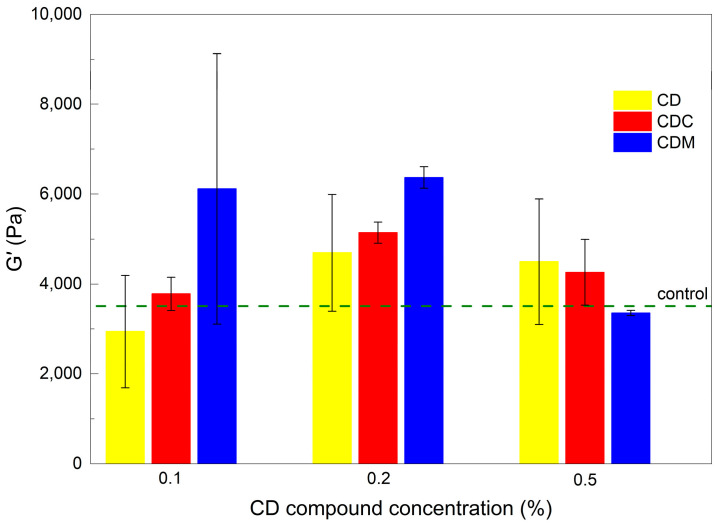
Final storage modulus (G′) of the gels as a function of CD type and concentration.

Figure 7 shows mechanical properties of the gels after 24 h. Hydrogels with native CD, low CDC content, or high CDM content were most similar to the control in terms of final storage modulus (G′), while other samples showed increased gel strength. Higher CD derivative content resulted in higher final G′ values, confirming the impact of both type and concentration of CD on not only the gelation kinetics but also the final mechanical stability of the hydrogel network.

All formulations exhibited typical gel-like behavior, although higher CD derivative content reduced the LVE range and mechanical robustness, highlighting the trade-off between flexibility and network stability.

##### Conclusion of Viscoelastic Gelation Studies

Strain-sweep tests showed that gels containing CD exhibited improved elasticity and resistance to deformation. The LVE was extended, and higher critical strain values were measured for some modified samples. This indicates improved mechanical integrity, potentially making these gels suitable for further application in controlled release systems.

Tests confirmed the formation of viscoelastic, solid-like networks in all gel samples with well-defined LVE regions. However, the extent of LVE and the critical strain at which the structure began to collapse varied depending on the type and concentration of CD used. Gels without CD tolerated higher strains before the structure collapsed, while gels containing native CD or randomly methylated CD had lower critical strains, indicating a more fragile network structure. These observations support the hypothesis that CDs can interfere with the ionic gelation process of alginate.

**Figure 8:**
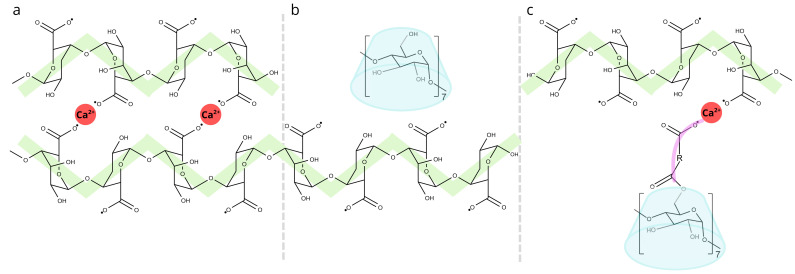
Schematic representation of alginate–CD interactions. (**a**) Alginate chains crosslinked via Ca^2+^ ions, forming the classical “egg-box” structure. (**b**) Native CD entrapped within the alginate matrix without direct bonding to the network. (**c**) Citric- or maleic-acid-modified CD (CD–R–COOH) interacting with alginate chains through Ca^2+^ bridges.

Overall, the viscoelastic measurements show that the alginate forms robust ionic gels when Ca^2+^ is added, while the inclusion of CDs modulates the gelation kinetics, network strength and mechanical stability (Figure 8). This modulation is likely due to absence, or presence and extent of non-covalent interactions between the CDs and the alginate chains or competition for calcium ions. These factors should be considered in the development of CD-functionalized hydrogels for biomedical applications such as controlled drug delivery, as this will likely affect gel porosity and diffusion properties.

In particular, the weak crosslinking and interactions, for example, van der Waals forces, hydrogen bonding and hydrophobic interactions, between the carboxyl groups of the CD derivatives and the alginate matrix probably interfere with stable gel formation during early gelation. In contrast, native CDs lacking acid species did not interfere with gelation. The citrate and maleate components in CDC and CDM, respectively, likely competed for Ca^2+^, forming chelates and reducing the availability of ions for alginate crosslinking and delaying gelation process.

The amplitude sweep tests also revealed that even though CD-containing hydrogels had higher storage moduli after 24 h and a narrower LVE, particularly for CDC-containing samples, this indicates the earlier structural failure under mechanical loading. At higher loading, CDC and CDM samples showed a faster decrease in G′, likely due to a higher number of weaker internal crosslinks. This reflects a trade-off between early viscoelastic strengthening and long-term mechanical robustness.

While lower concentrations of CD additives had minimal effects on gelation, higher concentrations (especially CDC_0.5_ and CDM_0.5_) delayed gelation, decreased network stability and increased energy dissipation under mechanical stress. These results highlight the need to optimize both type and concentration of CD derivatives for targeted applications.

### 2.4. Preparation and Characterization of Microbeads

Microbeads were produced to replicate bulk gel behavior in a scalable encapsulation format and to study how CD type affects morphology.

Alginate-based hydrogel microbeads were prepared with or without CD addition using an electrostatic vibration nozzle device to introduce microdroplets into a CaCl_2_ hardening solution (Table 2).

The average diameter of the obtained microbeads is in the range between 800 and 1000 μm. Incorporation of CD compounds influenced both the size and morphology of the obtained microbeads. Irregular or oval shapes were observed more frequently in CD-containing samples, probably due to changes in solution viscosity or surface tension during bead formation. A clear trend was observed as the diameter of the beads increased proportionally with the concentration of the added CD derivative (CD, CDC, or CDM). This increase in size is attributed to increased viscosity of the feed which has higher CD content, as confirmed in prior rheological observations, leading to the formation of larger droplets in the hardening solution.

CD incorporation increased bead size and irregularity in proportion to concentration, indicating viscosity-dependent droplet formation during vibration-assisted gelation.

### 2.5. Release of CD Components from Alginate Microbeads

Release experiments were conducted to evaluate CD retention and diffusion from alginate matrices, providing insight into network incorporation and potential controlled-release applications. The release experiments were not primarily intended to evaluate delivery performance but to quantify how much of the cyclodextrin component becomes structurally incorporated into the alginate matrix versus remaining unbound, thus having the ability to diffuse out of the hydrogel. This approach provides a means to assess the network integration efficiency and the stability of CD–alginate interactions under diffusion-controlled conditions.

The experimental design focused on elucidating the structural role of cyclodextrins within the hydrogel network rather than their carrier-release functionality. By monitoring CD diffusion from alginate microbeads, we aimed to determine the extent to which native and modified CDs participate in gel formation and remain entrapped in the ionic network. The fraction that diffuses out during incubation reflects the portion of non-integrated or weakly bound CD species, thereby providing indirect insight into the gel architecture and degree of network incorporation.

**Figure 9:**
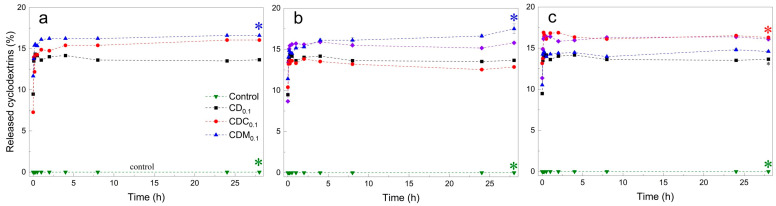
Release of different CD compounds from alginate-based microbeads over 28 h, at 0.1% (**a**), 0.2% (**b**), and 0.5% (**c**) inclusion levels in the hydrogel matrix. Statistically significant differences in release compared to the reference curve (CD_0.1_) are marked with *. Values are presented as mean ± SD.

To investigate the influence of CD structure on gelation and matrix incorporation, release studies were performed for all CD-loaded alginate microbeads over 28 h (1680 min) at 25 °C using UV–Vis spectrophotometry (Figure 9).

A high initial release of 7–13% was observed for all CD-loaded samples, probably due to partial diffusion of unbound CDs during the early gelation phase (the first ~5 min) when the polymer network was still forming. The burst release subsided within 2 h. Thereafter, negligible release was observed (cumulative release remained below 18% over 28 h), indicating that most of the CD derivatives remained within the hydrogel matrix as crosslinking/gel formation progressed.

At low CD concentrations of 0.1%, the highest cumulative release was observed for CDC_0.1_, followed by CDM_0.1_ and CD_0.1_. At 0.2%, the release was highest for CDM_0.2_, with no statistical difference between CD_0.2_ and CDC_0.2_. At the highest concentration (0.5%), release was highest at CDC_0.5_, followed by CD_0.5_ and CDM_0.5_. The final cumulative release after 28 h ranged from 13% for CDC_0.2_ to 18% for CDM_0.2_, with most formulations retaining more than 80% of the loaded CD. This is also strongly in line with the proposition that the CD release is occurring during gel formation [1].

These results indicate that the release kinetics were strongly influenced by CD type and concentration. The low release indicates efficient integration in the hydrogel network, likely due to ionic crosslinking and potential interactions between CD molecules and alginate chains. Despite the expectation that acid-functionalized CDs (CDC and CDM) would form additional crosslinks via carboxyl groups, the observed release patterns suggest that these interactions may be weaker than the hydrophilic–hydrophobic interactions between the CD cavity and the alginate chains.

In addition, samples with modified CDs showing greater variability in release indicate increased solubility and weaker retention in the matrix. Microstructural interactions, including possible chelation of Ca^2+^ by citrate or maleate groups, may have affected uniform crosslinking, resulting in greater release in the early phase. This was particularly noticeable for CDM_0.1_, CDM_0.2_ and CDC_0.5_.

The results confirmed that the majority (>80%) of CD derivatives were structurally retained within the hydrogel matrix after 28 h, demonstrating stable incorporation into the crosslinked network. The slightly higher release observed for CDC and CDM indicates a fraction of less-integrated molecules, consistent with their competitive interaction for Ca^2+^ ions and locally reduced crosslink density. These findings highlight that the designed system effectively immobilizes most of the CD component within the gel carrier rather than serving as a release vehicle.

## 3. Conclusions

This study demonstrates solvent-free modification of *β*-CD with CA or MA, affecting the gelling dynamics, viscoelastic properties, and release performance of CD–alginate hydrogels significantly.

Efficiency of functionalization: Solvent-free protocols yielded CDC and CDM derivatives with confirmed esterification and degrees of substitution (~4.0 for CDC, ~3.4 for CDM). These modifications improved water solubility and introduced reactive carboxyl groups.

Rheological behavior: Native CD accelerated gelation, while CDC and CDM delayed the process and decreased final gel strength, likely due to competition for Ca^2+^ ions and weaker network crosslinking. CDM at high concentrations produced the weakest hydrogel structures.

Mechanical properties: Amplitude sweep tests showed that gels with modified CDs had narrower linear viscoelastic ranges, indicating earlier structural deformation, although some formulations had an increased final storage modulus after 24 h.

Microbeads: Microbeads of 800–1000 µm were produced by vibrational prilling, increasing in size with CD concentration. CD incorporation influenced the morphology of the beads and surplus CD release.

Release behavior: More than 80% of CDs remained entrapped in the polymer network after 28 h, with slightly lower retention of CDC and CDM, compared to native CD.

### 3.1. Potential Applications and Future Directions

Based on their viscoelastic and mechanical properties, these gels are particularly suitable for biomedical and soft-material applications where controlled elasticity and deformation behavior are crucial, such as injectable hydrogels, wound dressings, soft-tissue scaffolds, and viscosity modifiers [44,45,46]. Although this work focused on understanding gelation mechanisms rather than developing an active delivery system, these findings provide a foundation for further studies on functional, biocompatible, and tunable hydrogel materials optimized for specific applications.

### 3.2. Limitations and Outlook

This work aimed to understand how cyclodextrin modification influences alginate gel structure and stability, rather than to develop an active delivery system. The main limitation is that techniques such as DLS provide only indirect information on nanoscale organization. Future studies should combine complementary analyses to visualize CD distribution and clarify how the degree of substitution and alginate composition jointly affect network behavior. Additionally, exploring lower CD and crosslinker concentrations could provide deeper insight into gelation dynamics, network development, and diffusion control, further improving the cost-effectiveness and sustainability of these formulations.

## 4. Materials and Methods

### 4.1. Materials

Sodium alginate (medium viscosity, from brown algae, viscosity 200–300 cps, Sigma-Aldrich, St. Louis, CA, USA), *β*-cyclodextrin (≥98%, Sigma-Aldrich), citric acid monohydrate (≥99.5%, Merck, Darmstadt, Germany), maleic anhydride (≥99%, Sigma-Aldrich), calcium chloride dihydrate (≥99%, Merck), and sodium phosphate (Na_2_HPO_4_, ≥99%, Merck) were used as received without further purification. Milli-Q ultrapure water (resistivity 18.2 MΩ·cm) was used for all preparations. Analytical balances (Kern ABP 200-5DM (Kern & Sohn GmbH, Balingen, Germany) and Exacta 2200 EB (Bio-optica s.p.a., Cinisello Balsamo, Italy)) were used for mass determination.

### 4.2. Synthesis of Modified CDs

Modified *β*-CDs were synthesized with CA and maleic anhydride via solvent-based and solvent-free methods, followed by purification and characterization.

CDC was synthesized using a modified protocol for CD-CA polymerization, based on [17]. *β*-CD (Sigma-Aldrich, USA) was functionalized with anhydrous CA (Sigma-Aldrich) in the presence of disodium hydrogen phosphate (Na_2_HPO_4_; Merck, Germany) as a catalyst. Various molar ratios of CD:CA:Na_2_HPO_4_ were tested, including 1:0.5:2.3, 1:1:2.3 and 1:2.3:2. Reactions were carried out at 100 °C, 120 °C or 140 °C for 10 or 30 min.

For lower reagent concentrations, mixtures were vortexed (Vortex Genius 3, IKA, Staufen, Germany) in 10 mL deionized water (15.5 MΩ·cm) and concentrated using a rotary vacuum evaporator (RVC 2-33 CD plus, Christ, Martin Christ, Osterode am Harz, Germany) at 80 °C and 1650 rpm. For more viscous mixtures (1:2,3:2), evaporation was not necessary, and the mixtures were reacted directly in the dryer. The dried products were redissolved in 10 mL of distilled water and centrifuged at 9000× *g* for 10 min (Rotanta 460R, Hettich, Andreas Hettich GmbH & Co. KG, Tuttlingen, Germany). Redissolution and centrifugation were repeated three times. Supernatants were filtered using Amicon Ultra centrifugal filters (3 kDa cutoff; Millipore, Burlington, MA, USA; 3 × 10 min at 5000× *g*), and both filtrates and retentates were freeze-dried (Alpha 1-2 LD plus, Christ, Osterode am Harz, Germany), homogenized and stored in a desiccator at 22 °C until further use.

CDM was synthesized by two methods.

Wet method: *β*-CD (3.41 g) and maleic anhydride (2.94 g; Sigma-Aldrich) were suspended in 6.0 mL of anhydrous DMF in a sealed high-pressure glass tube and heated at 70 °C for 18 h. The mixture was cooled and poured into 40 mL of chloroform to precipitate the product. The white precipitate was filtered, washed four times with 15 mL chloroform and dried on air to yield white powder in 123% yield (5.34 g) according to the substitution ratio of 3.2. The excess mass is attributed to impurities and DMF residue.

Dry method: *β*-CD (4.0 g) and maleic anhydride (4.1 g) were ground using a planetary ball mill (Fritsch Pulverisette 7, FRITSCH GmbH, Idar-Oberstein, Germany) at 500 rpm for 6 h at ~40 °C. After five replicates, the combined material was suspended in 50 mL of absolute ethanol, stirred for 2 h (IKA RCT digital, IKA-Werke GmbH & Co. KG, Staufen, Germany), filtered, washed three times with 20 mL of ethanol, and dried in air to yield white powder in 66% yield (17.0 g) according to the substitution ratio of 3.4.

### 4.3. Hydrogel Preparation

#### 4.3.1. Bulk Gels

##### Polysaccharide–Mineral Suspensions for Rheological Analysis

To investigate the gelling dynamics and viscoelastic properties, a series of polysaccharide–mineral suspensions were prepared. In the first series, 0.4 g of calcium carbonate (Sigma-Aldrich) was dispersed in 14 mL of distilled water, sonicated at 25 °C for 10 min, and combined with 0.4 g of a particular sodium alginate variant. The mixture was stirred overnight at 600 rpm. In the second series, 0.2 g of CaCO_3_ was dispersed in 1 mL of water, sonicated and supplemented with 0.2 g of sodium alginate and 13 mL of water, followed by the addition of filtered CD, CDC or CDM solutions (added all at once) at concentrations of 20, 40 or 100 mg. These suspensions were also stirred overnight to ensure complete homogenization, which was critical for reliable viscoelastic measurements. In each case, larger quantities were prepared to allow for consistent sampling during the rheological tests.

##### Viscoelastic Characterization of the Gels

The rheological measurements were performed using an oscillating rheometer (Anton Paar MCR 301, Anton Paar GmbH, Graz, Austria) with a parallel plate system (PP25) at 20 °C and a constant frequency of 1 Hz and controlled via the RheoCompass software (version 2.1, Anton Paar GmbH, Graz, Austria). The lower plate was fixed and the upper plate oscillated. The measuring surfaces were cleaned before and after each test to ensure reproducibility.

##### Gelation Monitoring of Alginate Formulations

To monitor the gelation kinetics, 1 mL of a prepared alginate-calcium mixture was added to the rheometer plate. The final concentrations were adjusted to 2% (*w*/*v*) alginate and calcium chloride and 0.75% (*w*/*v*) glucono-δ-lactone (GDL), which was previously dissolved and vortexed into the sample before loading. The sample was placed between the plates (gap: 1.5 mm; strain: 0.01%), and any excess was removed with a spatula. All rheological measurements were performed at 20 °C.

For the formulations with CDs (CD, CDC, CDM), the final concentrations were 1% (*w*/*v*) alginate and calcium chloride, 0.1–0.5% (*w*/*v*) CD and 0.375% (*w*/*v*) GDL. Control samples were prepared by adjusting the concentration of the mineral polysaccharide solution in the absence of GDL. Amplitude sweeps were conducted at 1 Hz (0.001–150% strain) after 24 h gelation. An amplitude sweep was performed before the measurements to ensure that the strain remained in the linear viscoelastic range. Gelation was monitored for 15 min. Preparation time, from GDL addition to the start of the measurement, was standardized (approximately 2–3 min) to ensure reproducibility.

##### Amplitude Sweep of Fully Gelled Hydrogels with CDs

For the amplitude sweep test, 3.5 mL of the finished hydrogel mixture (with 0.375% GDL) was poured into ring molds (approx. 65 mm high). The bottom was sealed with silicone grease, and the molds were covered with parafilm. The gels were measured after 24 h.

The samples were glued to foil-coated rheometer plates using cyanoacrylate adhesive. Amplitude sweeps at 1 Hz over a strain range of 0.001–150% were performed to evaluate mechanical stability (G′, G″). Control samples without CD were used for comparison.

#### 4.3.2. Microbeads

##### CD–Alginate Feed Preparation

For the preparation of polysaccharide solutions intended for microbeads preparation, either CD, CDC or CDM was weighed in amounts corresponding to 0.1%, 0.2% or 0.5% [*m*/*v*] (50, 100 or 250 mg) and added to 50 mL of Milli-Q water. The CD solutions were sonicated for 30 s in a Sonorex Super RK-52 ultrasonic bath (Bandelin, Berlin, Germany; 35 kHz, 60 W nominal power), magnetically stirred for 30 min (IKA RCT digital) until fully dissolved and subsequently filtered through a filter membrane with a pore size of 1 μm. Then, 1 g of sodium alginate powder (low, medium or high viscosity, Sigma-Aldrich) was added to obtain a 1% alginate solution. The mixture was sealed with parafilm and stirred for 16 h at 22 °C at 600 rpm.

##### Vibrational Prilling

Alginate-based hydrogel microbeads were prepared using a vibrating nozzle encapsulation system (Encapsulator B-395 Pro, Büchi, Flawil, Switzerland). Before each encapsulation run, all components of the device (except the vibrating membrane) were thoroughly cleaned in ultrapure water in an ultrasonic bath (Sonorex TK 52, Bandelin electronic GmbH & Co. KG, Berlin, Germany). The nozzle was pre-rinsed with distilled water to ensure proper flow and cleanliness.

Each prepared alginate–CD solution was filled into a 60 mL syringe and connected to the encapsulation apparatus. Operating parameters such as flow rate, vibration frequency and electrode voltage were individually optimized for each formulation. Adjustments were made in real time while the flow of droplet formation was observed under stroboscopic illumination. The aim was to obtain a stable, evenly distributed chain of droplets about a few centimeters below the nozzle tip.

The generated microbeads were collected in a glass beaker containing 250 mL of a 2% (*w*/*v*) calcium chloride dihydrate solution (CaCl_2_·2H_2_O, Sigma-Aldrich), which served as a crosslinking bath. The beaker was placed on a magnetic stirrer (IKA RCT Digital) set to 100 rpm to prevent aggregation of the beads during gelation. Once the entire volume of hydrogel had been extruded through the nozzle, the beads were allowed to cure in the crosslinking solution for a further 30 min with constant stirring.

The frequencies, voltages and flow rates used for each formulation are listed in Table 3. These conditions were chosen to ensure uniform morphology of the beads and optimal gel formation.

### 4.4. Characterization Methods

#### 4.4.1. Particle Size and Zeta Potential

Particle size and distribution were analyzed by dynamic light scattering (DLS) using a Zetasizer Nano ZSP (Malvern Panalytical, Malvern, UK) at 25 °C. Zeta potential was determined by laser Doppler electrophoresis in with a barrier diffusion method with a deposit of 20 µL sample on the bottom of a folded capillary cell, pre-rinsed with pre-filtered (d_f_ = 0.22 µm) ethanol and water and filled with a 0.05% aqueous NaCl solution.

#### 4.4.2. FTIR—Fourier Transform Infrared Spectroscopy

FTIR spectra were recorded using a PerkinElmer Spectrum Frontier IR spectrometer (version 10.6.2, PerkinElmer, Inc., Waltham, MA, USA) with an ATR diamond crystal. The samples were placed directly on the crystal, and the spectra were recorded between 4000 and 450 cm^−1^ at room temperature. The ATR crystal was cleaned with isopropanol after each sample.

#### 4.4.3. ^1^H Nuclear Magnetic Resonance (NMR)

Proton NMR spectra were recorded using a Bruker Avance III UltraShield 500 Plus spectrometer (Bruker, Billerica, MA, USA) at 500 MHz and 25 °C. CD derivatives were resolved in D_2_O or DMSO-d_6_ depending on solubility. Chemical shifts were calibrated using internal tetramethylsilane (TMS), and data were processed using MestReNova software (version 14.3.1, Mestrelab Research, Santiago de Compostela, Spain).

#### 4.4.4. Light Microscopy

Morphological analysis of the alginate-based microbeads was performed using a Leica DM750 light microscope (Leica Microsystems GmbH, Wetzlar, Germany) at 40× magnification. To transfer the microbead suspension, the tip of the plastic pipette was cut, and a few drops were placed on the microscopy slide. Multiple micrographs were recorded per sample with the Leica LAS EZ software (version 3.4.0, Leica Microsystems, Wetzlar, Germany). Size analysis was performed on at least 50 beads per sample with the FIJI ImageJ2 software (ImageJ2, version 1.53t, National Institutes of Health, Bethesda, MD, USA).

#### 4.4.5. Quantification of Released CDs by UV–Vis Spectrophotometry

The release of native CD, CDC and CDM from alginate-based hydrogel microbeads was monitored for 28 h using UV–Vis spectrophotometry (UV-8453, Hewlett Packard, Palo Alto, CA, USA). The method was adapted from [47] and is based on the decrease in absorbance of free phenolphthalein at 550 nm due to the formation of inclusion complexes with released CDs. The complex formation reduces the intensity of the pink coloration of phenolphthalein in alkaline medium and allows for a quantitative evaluation of the released CD derivatives.

A 3 mM phenolphthalein stock solution was prepared by dissolving 9.55 mg phenolphthalein (Kemika, Zagreb, Croatia) in 10 mL 70% [*v*/*v*] ethanol. The working solution was prepared by mixing 1.0 mL of the stock solution with 98 mL of freshly prepared 0.125 M glycine buffer (0.938 g glycine in 100 mL water), the pH of which was adjusted to 10.50 with the 10 M NaOH solution. The final solution was mixed until homogeneous, transferred to a sealed glass vial and wrapped in aluminum foil to prevent photodegradation of the indicator. Calibration curves for CD, CDC and CDM were prepared using stock solutions (2.724 mg/mL in 2% CaCl_2_·2H_2_O, Sigma-Aldrich) and diluted to six calibration points (0.0664–1.362 mg/mL). Each sample (0.2 mL) was mixed with 1.0 mL of phenolphthalein working solution and the absorbance at 550 nm was recorded immediately (UV-Vis spectrophotometer, HP 8453, Hewlett Packard, Palo Alto, CA, USA). The absorbance differences were calculated in comparison to a control, and the resulting data were fitted using either Michaelis–Menten equations or fourth degree polynomial equations (R^2^ > 0.999).

The release of CDs from alginate microbeads was monitored by taking 0.5 mL aliquots from the 2% CaCl_2_ crosslinking solution at different time points up to 28 h. CD-free controls were included. The concentrations of released CD, CDC and CDM were calculated using the corresponding calibration curves.

The stability of the phenolphthalein indicator was checked by measuring its absorbance over 28 h. The working solution was prepared by combining 2.0 mL of 3 mM phenolphthalein in 70% ethanol (Merck) with 98 mL of glycine buffer (0.125 M, pH 10.5, adjusted with 10 M NaOH, Kemika) and stored in light-protected containers.

### 4.5. Data Processing and Statistical Analysis

All data processing and visualization were performed using OriginPro software (Origin 2018, version 9.5, OriginLab Corporation, Northampton, MA, USA). For graphical representation, release profiles and rheological measurements were smoothed with adjacent averaging. Where applicable, averaged datasets were fitted with 2nd–5th order polynomials to visualize overall trends. Polynomial fits were used exclusively for data presentation and not for statistical inference.

To compare the similarity of the release profiles of CD, CDC and CDM from alginate microbeads into the crosslinking solution, the similarity factor (f_2_) was calculated in accordance with FDA guidelines [48]. An f_2_ value between 50 and 100 indicates the similarity between two profiles. All profiles were compared to the reference formulation CD_0.1_.

## Figures and Tables

**Table 1:** Linear viscoelastic range and modulus crossover points from amplitude sweep tests.

Sample	Critical Strain *γ*_c_ (%)	Modulus Crossover Point (%)	Sample	Critical Strain *γ*_c_ (%)	Modulus Crossover Point (%)	Sample	Critical Strain *γ*_c_ (%)	Modulus Crossover Point (%)
CD_0.1_	0.068	111	CDC_0.1_	0.045	111	CDM_0.1_	0.089	111
CD_0.2_	0.039	111	CDC_0.2_	0.039	128	CDM_0.2_	0.068	97
CD_0.5_	0.059	128	CDC_0.5_	0.045	128	CDM_0.5_	0.015	74
Control	0.110	111	Control	0.110	111	Control	0.110	111

**Table 2:** Size distribution (µm) of alginate-based hydrogel microbeads containing CD, CDC, and CDM. The annotated sample labels indicate CD compound concentration (% *w*/*v*) in the microbeads. Optical micrographs were obtained under 40× magnification.

	CD_0.1_	CD_0.2_	CD_0.5_
Mean	867	903	972
Standard deviation	112	140	171
Median	867	907	949
Minimum	616	652	665
Maximum	1137	1329	1520
Images	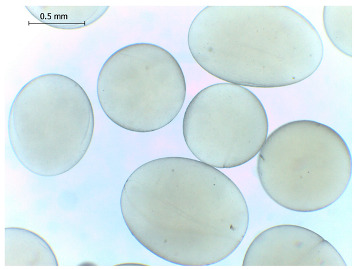	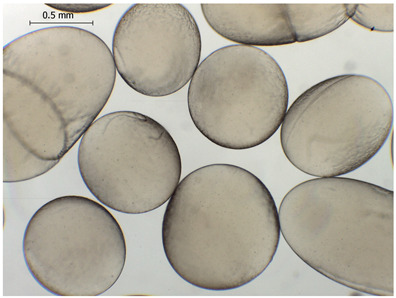	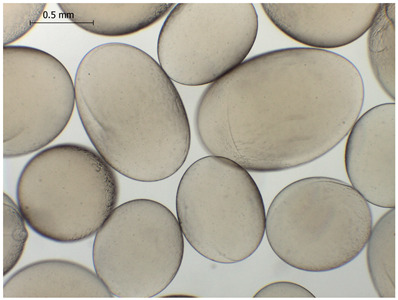
	CDC_0.1_	CDC_0.2_	CDC_0.5_
Mean	836	916	929
Standard deviation	132	127	131
Median	789	918	910
Minimum	669	592	690
Maximum	1231	1149	1291
Images	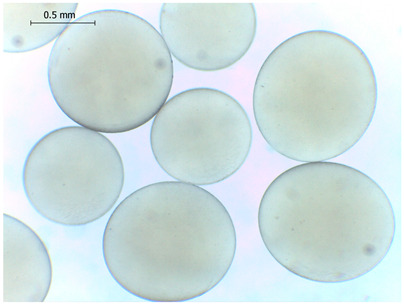	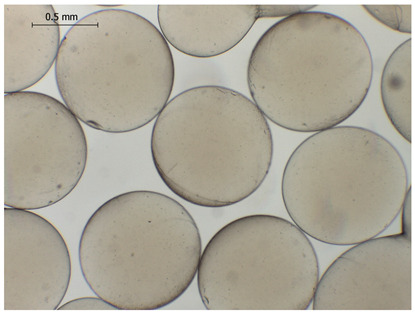	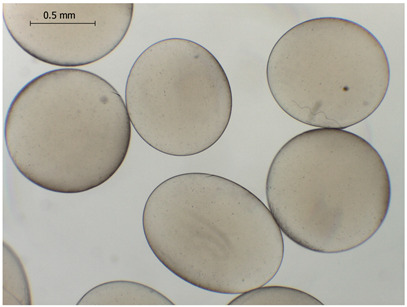
	CDM_0.1_	CDM_0.2_	CDM_0.5_
Mean	813	801	823
Standard deviation	133	122	142
Median	775	758	758
Minimum	618	615	622
Maximum	1101	1291	1375
Images	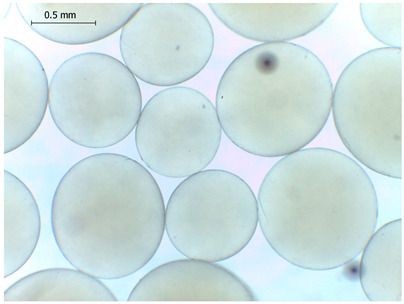	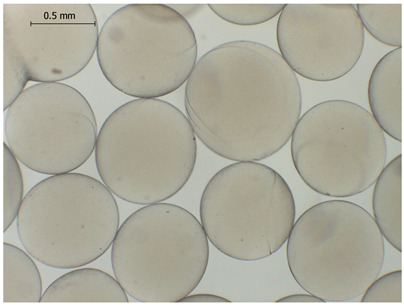	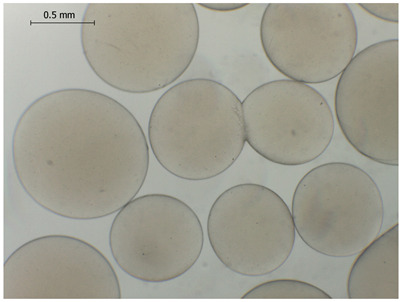
	Control		
Mean	861		
Standard deviation	134		
Median	861		
Minimum	637		
Maximum	1238		
Images	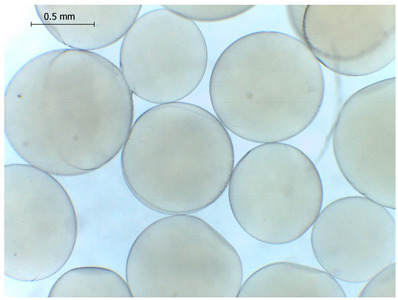		

**Table 3:** Overview of the optimized operating parameters for microbead preparation.

	Frequency (Hz)	Voltage (V)	Flow Rate (mL/min)
Alginate without CD	1100	1100	10
CD_0.1_, CD_0.2_, CD_0.5_	1100	1100	10
CDC_0.1_	1100	1100	8
CDC_0.2_	1100	1100	9
CDC_0.5_	1100	1100	9
CDM_0.1_	1300	1100	8
CDM_0.2_	1300	1100	8
CDM_0.5_	1300	1100	8

## Data Availability

The original contributions presented in this study are included in the article/Supplementary Material. Further inquiries can be directed to the corresponding author.

## Supplementary Materials

The following supporting information can be downloaded at: https://www.mdpi.com/article/10.3390/gels11110875/s1, Figure S1: Particle size distribution of the dialyzed filtrate sample VZ2-30 measured in 0.05% (*w*/*v*) NaCl solution. Three parallel measurements are shown (curves in different colors). PDI –polydispersity index; Figure S2: ^1^H-NMR spectra of (a) CA-modified *β*-CD (b) MA-modified *β*-CD obtained via solvent-based method (c) MA-modified *β*-CD obtained via solvent-free method. Chemical shift (δ, ppm) is shown on the *x*-axis, and signal intensity is on the *y*-axis. Figure S3: Mass spectrum of the main functionalization product of CK and CD in positive ionization mode (*x*-axis: mass-to-charge ratio, *m*/*z*; *y*-axis: signal intensity); Figure S4: Storage modulus (G′) and loss modulus (G″), plotted on a logarithmic scale, of 1.5% sodium alginate solutions containing different concentrations of differently modified cyclodextrins (CDX), where X is: (a) native CD, (b) citric-acid-modified CD, and (c) maleic-acid-modified CD, measured prior to gelation without the addition of the crosslinking agent GDL. Linear regression curves with 95% confidence intervals are shown to illustrate the viscoelastic development over time. Values below 1 Pa are not shown on the y-axis for G′ and G″, as they fall below the reliable detection limit; Table S1: Zeta potential (mV) of filtrate and retentate fractions obtained from the first and second solvent-free modification experiments of β-cyclodextrin with citric acid (CDC). In the sample label (VZ), the first number indicates the molar ratio of citric acid to cyclodextrin (CA:CD = X:1, where X denotes the molar ratio), and the second number refers to the reaction time (in minutes) or temperature (in °C) of the dry-state modification.
