# Supplementary material for: Sustainable Cyclodextrin Modification and Alginate Incorporation: Viscoelastic Properties, Release Behavior, and Morphology in Bulk and Microbead Hydrogel Systems
Source: Gels. 2025 Nov 1;11(11):875. doi: 10.3390/gels11110875 (PMC12652062; doi:10.3390/gels11110875)
Supplement: Supplementary file 1 [file gels-11-00875-s001.zip › gels-3916684-supplementary.pdf]

Sustainable Cyclodextrin Modification and Alginate Incorporation: Viscoelastic Properties, Release Behavior, and Morphology in Bulk and Microbead Hydrogel Systems

Supplementary Materials

Maja Čič [1], Nejc Petek [2], Iztok Dogša [3], Andrijana Damjanović [3], Boštjan Genorio [2], Nataša Poklar Ulrih[3], and Ilja Gasan Osojnik Črnivec [1,3*]

[1]National Institute of Chemistry, Ljubljana, Slovenia
[2]Faculty of Chemistry and Chemical Technology, University of Ljubljana, Ljubljana, Slovenia
[3]Biotechnical Faculty, University of Ljubljana, Ljubljana, Slovenia
[*]Correspondence: gasan.osojnik@ki.si

1. Particle size and zeta potential

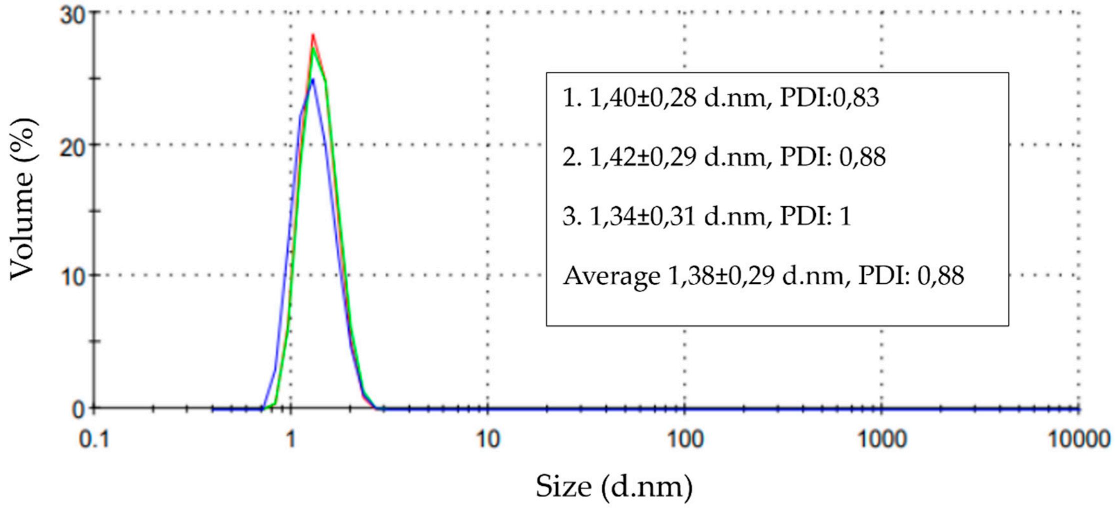

Figure S1: Particle size distribution of the dialyzed filtrate sample VZ2-30 measured in 0.05% (w/v) NaCl solution. Three parallel measurements are shown (curves in different colors). PDI –polydispersity index.

Table S1: Zeta potential (mV) of filtrate and retentate fractions obtained from the first and second solvent-free modification experiments of β-cyclodextrin with citric acid (CDC). In the sample label (VZ), the first number indicates the molar ratio of citric acid to cyclodextrin (CA:CD = X:1, where X denotes the molar ratio), and the second number refers to the reaction time (in minutes) or temperature (in °C) of the dry-state modification.

Sample	Dialysis					
	Filtrate 1 (mV)	Retentate 1 (mV)	Filtrate 2 (mV)	Retentate 2 (mV)	Filtrate 3 (mV)	Retentate 3 (mV)
VZ05-10	-25,5	-17,0	/	/	/	/
VZ1-10	-13,8	-15,4	/	/	/	/
VZ2-10	-7,1	-9,2	/	/	/	/
VZ05-30	-27	-20,1	/	/	/	/

VZ1-30	-17,4	-28,9	/	/	/	/
VZ2-30	-12,3	-18,8	-10,7	-18,3	-10,7	-15,5
VZ2-120	-5,8	-5,1				
VZ2-100	-5,3	-7,7				

2. ^{1}H NMR

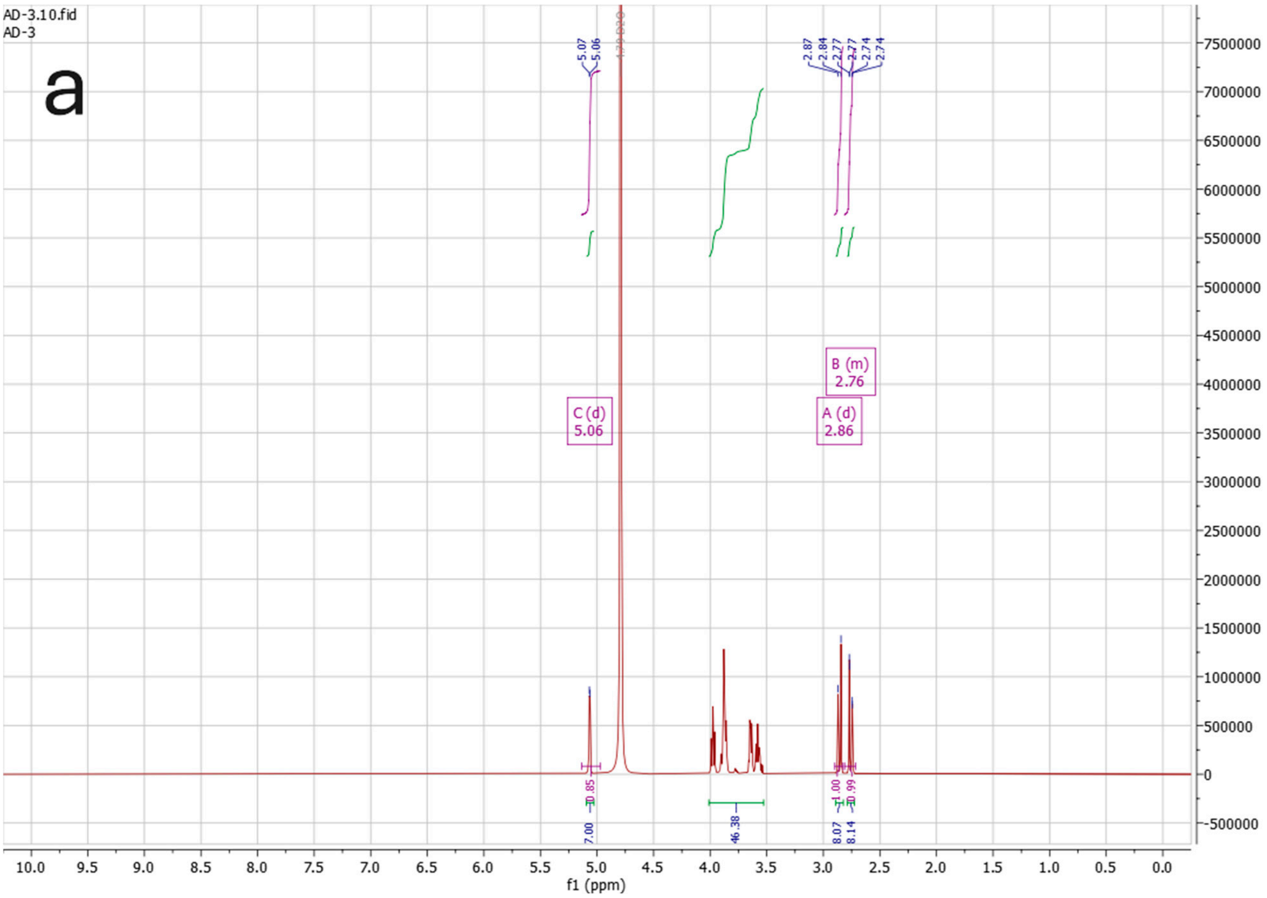

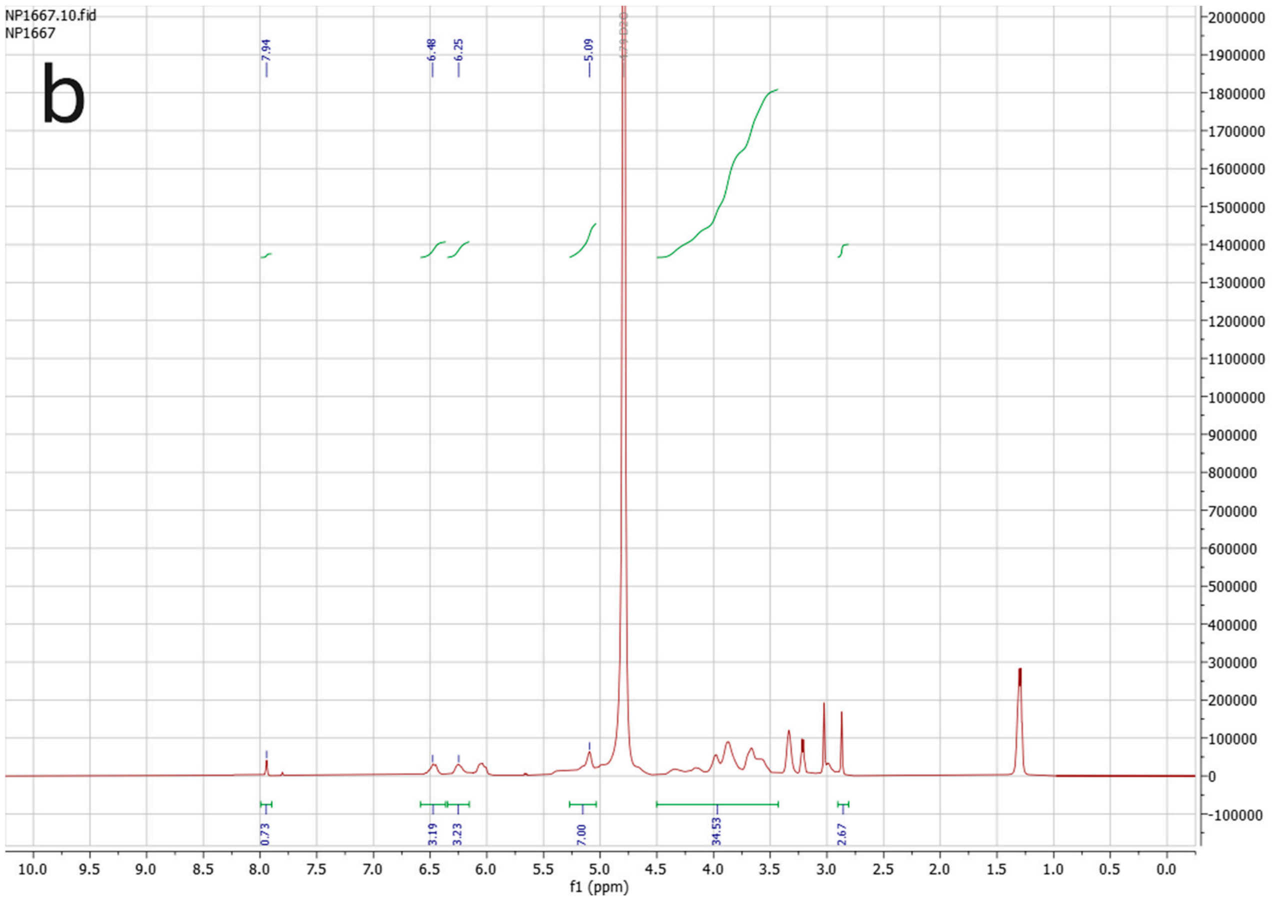
NP1667.10.fid
NP1667
b
7.94
6.48
6.25
5.09
0.73
3.19
3.23
7.00
34.53
2.67
10.0 9.5 9.0 8.5 8.0 7.5 7.0 6.5 6.0 5.5 5.0 4.5 4.0 3.5 3.0 2.5 2.0 1.5 1.0 0.5 0.0
f1 (ppm)
2000000
1900000
1800000
1700000
1600000
1500000
1400000
1300000
1200000
1100000
1000000
900000
800000
700000
600000
500000
400000
300000
200000
100000
0
-100000

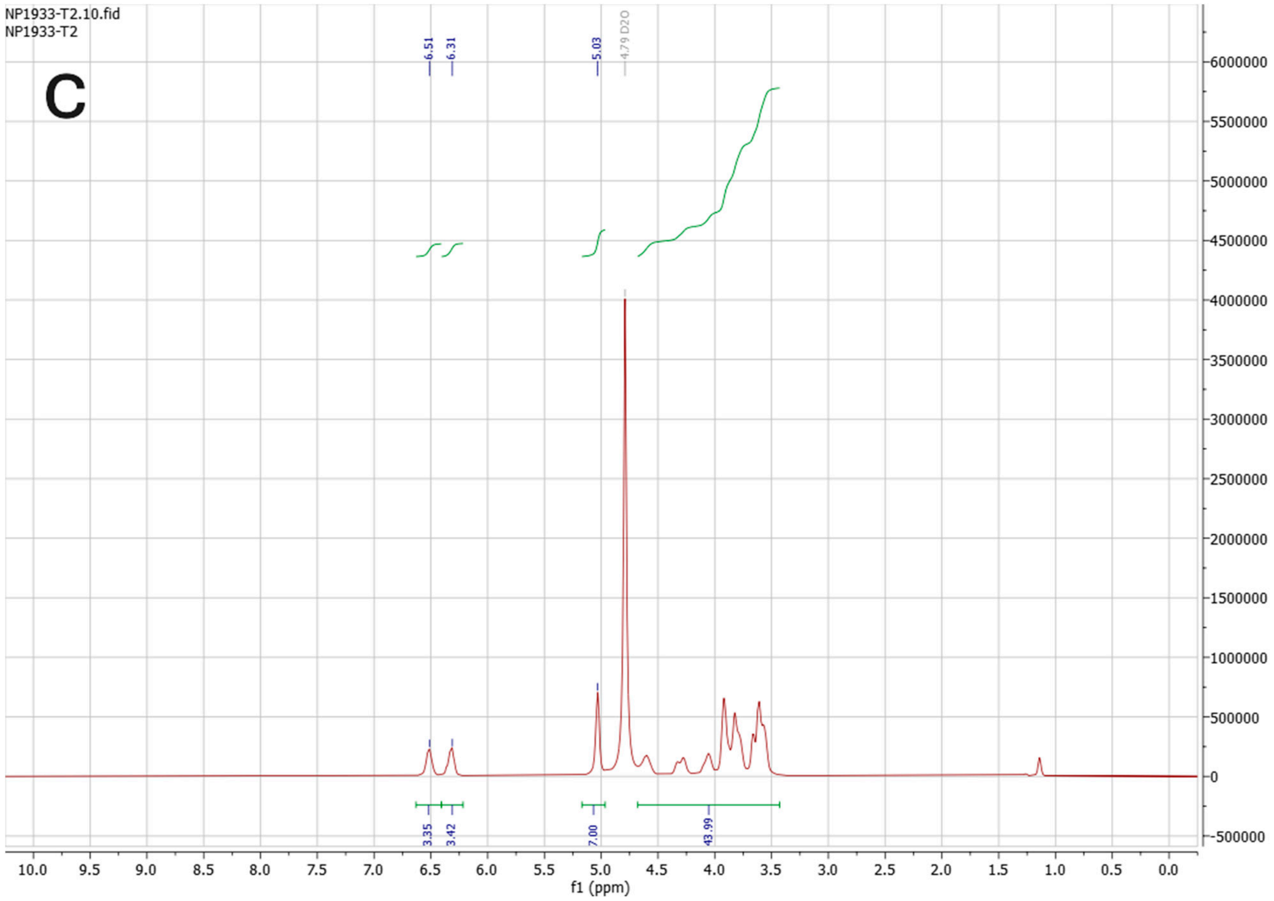

Figure S2: ¹H-NMR spectra of (a) CA-modified β-CD (b) MA-modified β-CD obtained via solvent-based method (c) MA-modified β-CD obtained via solvent-free method. Chemical shift (δ, ppm) is shown on the x-axis, and signal intensity is on the y-axis.

3. MS - mass spectrometry

Molecular weights and fragmentation patterns were determined by LC-MS on an Agilent 6224 TOF LC/MS system. CDC samples were dissolved in acetonitrile and analyzed at 25 °C.

Mass spectrometry (MS) is an efficient analytical method used for characterizing reaction products due to its high sensitivity, speed, and low sample requirement [26]. MS analysis was used to confirm the formation of cyclodextrin–citric acid conjugate (CDC) and to assess the stoichiometry between CD and CA.

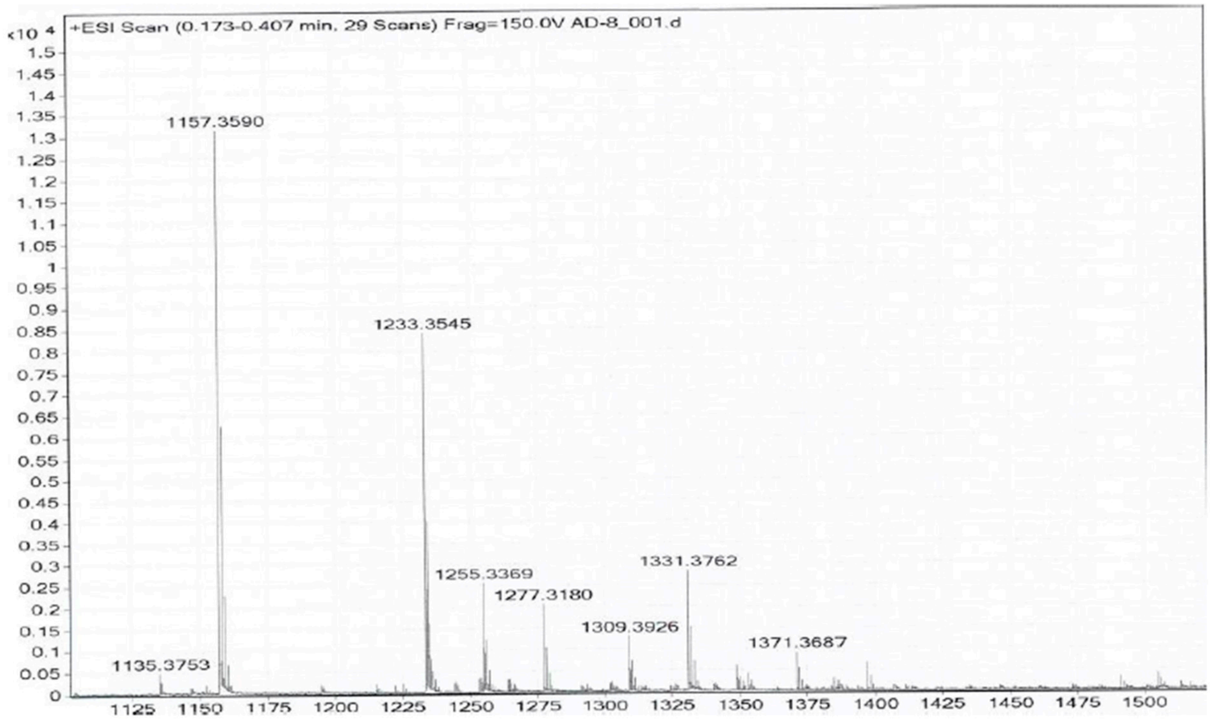

Figure S3: Mass spectrum of the main functionalization product of CK and CD in positive ionization mode (x-axis: mass-to-charge ratio, m/z; y-axis: signal intensity).

Positive ion mode mass spectrum (Figure S3) exhibited a peak at 1135.3753 m/z, corresponding to residual unmodified CD (HMDB). A distinct molecular ion peak at 1309.3926 m/z indicates the formation of CDC conjugate, confirming a 1:1 molar ratio of CD to CA (one substitution per CD molecule). These findings are consistent with our FTIR and NMR results and with literature data obtained via NMR [26].

Further MS analysis revealed additional peaks corresponding to mono-, di-, and tri-substituted CD-CA conjugates. These oligomeric species provide molecular-level confirmation of successful CD functionalization and allow estimation of the degree of substitution. Together with FTIR and NMR data, these results validate the covalent attachment of CA to CD and support the structural identity of the synthesized CDC products.

4. Alginate and cyclodextrin solutions

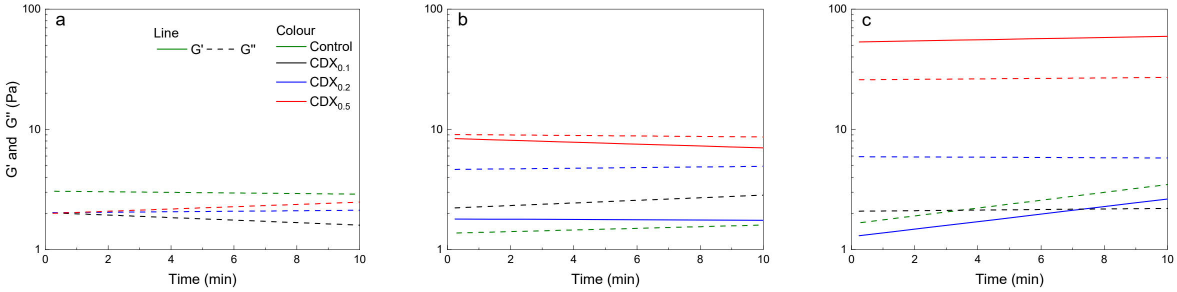

Figure S4. Storage modulus (G′) and loss modulus (G″), plotted on a logarithmic scale, of 1.5% sodium alginate solutions containing different concentrations of differently modified cyclodextrins (CDX), where X is: (a) native CD, (b) citric-acid-modified CD, and (c) maleic-acid-modified CD, measured prior to gelation without the addition of the crosslinking agent GDL. Linear regression curves with 95% confidence intervals are shown to illustrate the viscoelastic development over time. Values below 1 Pa are not shown on the y-axis for G′ and G″, as they fall below the reliable detection limit.

Figure S4 shows the storage modulus (G′) and loss modulus (G″) of alginate A033 blends with different CD derivatives in the absence of the crosslinking agent GDL. These measurements provide information about the basic viscoelastic properties of the systems before gelation is triggered.

In Figure S4(a), G″ dominates over G′ for all four samples tested, reflecting the viscous nature of the uncrosslinked blends. The negligible G′ values confirm the absence of an elastic or structured network. Of note, the addition of CD at different concentrations did not significantly affect the viscoelastic profile, indicating limited interaction at this stage.

Figure S4(b) shows the viscoelastic moduli of alginate solutions with different concentrations of CDC. A concentration-dependent increase in both G′ and G″ was observed, with $CDC_{0.5}$ showing the highest values, followed by $CDC_{0.2}$, $CDC_{0.1}$ and the control. This indicates that CDC increases the viscosity and elasticity of the solution before gelation.

In Figure S4(c), the samples were evaluated with CDM. Sample $CDM_{0.5}$ showed a notable difference: G′ exceeded G″, indicating the presence of a weakly structured gel-like network even without external cross-linking. In contrast, the other CDM-containing samples remained in a primarily viscous state (G″ > G′), consistent with the behavior of uncrosslinked polymer solutions.
